# Miscellaneous Paragraphs

**Published:** 1893

**Authors:** 


### PAINS AND THEIR SIGNIFICANCE.

*Of the Head.*—Headaches are very often due to affections of the eyes, such as weakness seen in convalescence from debilitating sickness; inherited weakness ; straining of the muscles of the eye; anatomical or other imperfections in the mechanism of sight; overuse, strain or abuse resulting from reading by bad light; dusty and irritating atmospheres, etc., etc. Dyspepsia, liver troubles, and constipation give rise to "sick headache," a dull aching pain, accompanied by more or less nausea. Constipation in these cases is generally an indication of the cause, and of a method of relief. Neuralgic pains are sharp, shooting, and almost unbearable ; generally a tender spot will be found in the vicinity. The pain follows the course of the nerves affected, a common location being just above one or both eyes, the tender point being just at the bony margin beneath the eyebrow. Nasal catarrh and inflammatory affections of the ear (often associated) are common causes of headache.

*Abdomen.*—Severe pains following closely upon a meal or the ingestion of other substances may result from colic, poisoning, inflammation, cancer or ulcer of the stomach. When paroxysmal and recurring at varying intervals, lead poisoning, neuralgia or rheumatism may be suspected. The lead poisoning may be the result of drinking water carried through lead pipe, or boiled in vessels containing lead; canned fruits, the use of hair dyes or cosmetics, and occupation involving the use of lead, as painters, tinsmiths, plumbers, etc. Pains high up and associated with a feeling of distress, distension, weight and eructation of gas and sour material indicate indigestion. Pains low down in the abdomen are found in women due to disorders and diseases peculiar to the sex, and are often combined with a feeling of weariness, pains in the back, and lassitude upon rising and retiring. Pains in the back, on each side, about the waist line, are significant of congestion or other trouble with the kidneys, rheumatism or neuralgia.

*Chest.*—When associated with soreness upon pressure located in the middle of the chest, just below the base of the neck, and in breathing at same point, bronchitis is the trouble, and affection of the larger lung tubes. If these symptoms are in the side and the pain especially marked in inspiration, the preferring to lie on the affected side, pleurisy. Pain in the side, hurried and difficult respiration, with reddish or rusty colored expectoration, probably pneumonia. Neuralgic pains are not associated with fever, generally follow the direction of the spaces between the ribs, and at some point along their course tender spots will be found, one generally close to the spine.

*Limbs.*—Pains in the legs or arms not dependent upon some sprain or injury, are generally rheumatic or neuralgic in their nature, and often both combined. Rheumatic pains are more often confined to the joints and associated with tenderness, swelling and redness in addition. Neuralgic pains are sharper, more paroxysmal in character, and follow the direction of the limb.

### ABOUT THE TEETH.

Very few people have clean teeth. At least that is what a dental surgeon says, and as he has many more patients than he can treat without a previous engagement, he is in a position to know a great deal about the subject. Brushing the teeth does not always cleanse them, according to this authority—that is, the hasty application of the brush. To be of service the brush should be oval in shape, quite small, absolutely without decoration, and of a long, fine and medium stiff bristle, so as to reach into every space and dislodge every particle of food.

It is of more importance that the inside of the teeth should be brushed than the outer side, as the accumulations are to be found there that cause not only bad breath but the destruction of the dentine. The motion of the brush should be up and down. Frequent or long brushing is not good, as the gums are apt to recede from the torture of stiff bristles.

Warm water is the best both for washing and rinsing the mouth. Warm soap suds made of palm-oil soap and used twice a week, is not agreeable, but the results are excellent ; being an antiseptic, the soap dissolves the deposits on the teeth and refreshes the whole mouth as few tooth powders can. After this, the only sure way of knowing that the teeth are clean is to pass a silk thread between them and finish with another gargle or rinsing of warm water. The use of a pin, needle or penknife as a toothpick is painful to think of, and an excellent way to ruin the teeth.

Many people lose their teeth by carelessness is eating or drinking very hot or cold substances, and also in taking very powerful drugs. Whenever possible liquid medicines should be taken from a medicine spoon and acidulous drinks through a straw. Perfect health is impossible without good teeth. Another reason for their preservation is the beauty they give the mouth. Loss of teeth puts hollows in the cheeks and makes the face old. A tooth should never come out while there is a nerve in it. Composition fillings are now not only inexpensive, but considered by the profession better than the finest gold. They do not wear as long, but neither do they require as much excavation as metal filling. Good work will last from one to two years and a small outlay will renew it.

### OVERWORK AND UNDERWORK.

Every one has heard of the danger of overwork, yet few understand just where the danger lies. A man can hardly overwork himself if he takes cares of himself in other respects—secures a normal amount of sleep, breathes pure air, takes exercises and eats food moderately.

The main trouble is that the man who is overworking is violating the fundamental conditions of health. He burns his candle at both ends.

With due care, a man of good heredity is capable of safely doing an almost incredible amount of solid work. Mr. Gladstone, at 83, with no show of weariness, carries the weight of the British Empire. The celebrated John Wesley did more work than almost any other man of the last century ; but he observed the laws of health, and still active, reached his 88th year.

Much of the so-called overwork is the work of worry, care, anxiety and haste. These make the severest draft on the vitality of the system.

We seldom hear of a Quaker's dying of overwork, and yet they are a very industrious people. The pupil who has prematurely broken down in his studies might have gone on under even heavier loads if there had been nothing to fret him in his home surroundings, and competition, examinations and scholarship markings had no place in our school system. The fact is, work, and plenty of it, is healthy in a high degree.

And this leads us to say that a lack of work, with brain or hand, is highly injurious. Underwork may be as harmful as overwork to the brain if not to the body. Nations living in conditions in which the means of livelihood come almost without effort are in every way feeble. Close confinement in prison tends to idiocy.

Further, where the mental faculties are not called into action, the moral also lie dormant, and the lower propensities become all-controlling. In all ages the corruptions of the higher classes are due to this fact. Few worse things can befall one than to have nothing to do.—*Youth's Companion.*

---

### A "BED DAY" FOR TIRED ONES.

It is told of Phœbe Cary, who was a remarkably sunshiny and loving woman, that whenever she used to feel "out of sorts," she would shut herself up in her room for rest until the serenity of soul was restored. She was wise enough to discern the physiological side of amiability and govern herself accordingly. A mother of two restless children acts upon the same principle by insisting upon an occasional "bed day." She has observed that "crossness" with them is invariably the result of too much activity; that nervous force is expended faster than muscular strength is generated, and tries to restore the balance in the manner suggested.

The children understand that the measure is not a punishment, but enforced solely for sanitary reasons, and are allowed plenty of playthings and quiet games. This mother testifies that the next day her small brood is "bright and chipper as young robins." The experiment for either children or adults is worth trying, especially after the excitement and irregular habits necessarily connected with a holiday season.

---

### FRUIT DIET.

Pimples, eruptions, and similar skin diseases of the face, that are not hereditary, may be cured in a very short time by a diet of laxative foods, varied according to the season. I advise ornamenting the table at each meal with whatever fruit is seasonable, and allowing the individual to be helped whenever and as often as he or she may desire. This serving the fruit course at the end of the meal, when the appetite is appeased to repletion, is a great mistake.

If I feel like eating an orange or a handful of dates, I do so, whether the soup has been served or not. When the dessert comes on the chances are even that I won't want any. That's my gain, not loss, for if I hadn't eaten the fruit I should have taken a dish of ice-cream, which little more than cools the mouth, and I would have had no room for the wholesome orange or apple. Chicken salad and patties, cheese, pastry, and a number of other popular and indigestible dishes, should have their turn at the end of the meal. The nearer the beginning such things as fresh ripe berries, juicy tropical fruits, asparagus, cauliflower, onions, beets, mutton, rare juicy beef, spinach, lettuce, squash and stewed seed fruits, such as figs, plums, prunes and cranberries are served, the better.

### DON'T SLEEP IN LINEN.

The truth about linen is that it isn't the ideal dressing for beds at all. It is cold and slippery and insures sensitive persons the dream of sleeping on an iceberg, which does well enough for an occasional experience, like seasickness, but which palls on too frequent repetition. Besides that, it wrinkles and tumbles, in spite of its heavier body, much more than cotton does, giving a bed after one night's use a most slovenly and uninviting appearance.

Nobody recommends linen for body wear. Its firm texture and hard surface make it wholly non-absorbent; it allows the body to become chilled by refusing the perspiration, and so has been known to bring on serious illness. For outside wear in summer, linen may be tolerated as clothing, but nowhere else. Where, however, it is at its most useful and best, is in household uses. For table service, for the toilet, and for minor ornamental details, it is simply invaluable; its smoothness of texture, its brilliancy, which laundering even increases, its exquisite freshness, makes it the one fabric fit to drape the dining table and to use in the toilet, while its suitability for needlework decoration makes it admirable for all kinds of fancy work. And here it is rightfully used, but to wear next the skin and sleep in—no.

### HOW SHE KEEPS WELL.

I have no time to be ill, so I take time for the little precautions which keep one well. I am always rather hurried in the morning, but not enough so to omit my bath and take cold. If, unfortunately, I have taken cold, I know I shall have no time to coddle influenza or bronchitis, so I make myself a hot lemonade, and put an extra covering on the bed at night. Then in the mornings I throw off coverings gradually, until I am cool enough to venture to rise to take the cold bath. That is nearly sure to arm me against further damage. Then, too, I can't give up a day every week or fortnight to nervous headache, so I must give an hour every day to walking in the open air. I try to keep my small belongings in order, and put each thing back in the place I took it from, so I don't wear out my nerves hunting for the button hook or hair curler. I can't stop the machinery, so I try to keep it in running order.

It is well to know, however, that a cold bath in the morning may benefit a young and strong constitution, but is injurious to weak persons and the aged.—*Selected.*

### WHEN THINGS GET IN THE EYE.

One of the most frequent and most annoying of the smaller accidents which are happening to us every day is the getting of small particles of dust and cinders in the eye. What is at first a loose attachment of such a body soon becomes a firm one by the rubbing of the afflicted eye which is sure to follow. When this happens to a child try to make him understand the rubbing only makes matters worse, and that it is best to let the free flow of tears called forth by the presence of the irritant wash it out. When this is not effectual, grasp the upper lid by the lashes and pull it well down over the lower lid, allowing it to sweep back over this part, thus cleaning it out. Most foreign bodies get entangled in the upper lid, so that this proceeding is usually effectual if such body is not deeply and firmly attached. If the body still remains, the lids must be everted over a pencil, and all parts, including the ball of the eye, be carefully examined in a good light. The disagreeable sensation may remain several hours or longer after the body has been actually removed, from the irritation already set in.

This can be palliated by freely bathing the lids with very hot water, holding a sponge so saturated over the closed eye.

### MILK AS A FOOD.

Have you fallen into error in regard to the nutritiousness of milk? A large number suppose it to be a good thing for children, but hardly for adults. As a matter of fact, there is no one article of diet, perhaps, which affords more nourishment and is more easily digested than milk. The digestion of milk is very simple. When it reaches the stomach, a selection of its directly nutritive principles begins. The albumen and butter solidify and coagulate, while the sugar remaining dissolved in the whey, passes into the bowels and may there at once be absorbed unchanged. This absorption involves no waste to nutritive substances, meanwhile the albumens or principals are absorbed without leaving any residuum. The same is true of the butter, and even to a larger degree, and it is the most digestible of all the fatty bodies. Unless there is some well founded reason against a milk diet, it will be found a very valuable article of food. With some persons hot milk is best, with others cold, while still others do best on skimmed milk. It is one of our most valuable articles of food.

### TO CURE A COUGH.

There are few disorders more teasing to the sufferer and to those about him than a cough. A slight hacking cough is often a bad habit; when it is at all under the control of the will, it should be sternly repressed. Sometimes the uvula, the pendulous part of the soft palate, at the back of the mouth, becomes relaxed, the point touches the tongue, producing a tickling sensation, which requires a cough to relieve it. A little dry tannic acid put in a quill and blown on the uvula will contract it, or half a teaspoonful of powder mixed with two teaspoonfuls of glycerine, stirred into half a glass of warm water and used as a gargle. When a cold has been taken and there is cough with soreness of the chest, bed should be prescribed for fear of a severe attack of bronchitis. Soak the feet in a pail of hot water in which is dissolved three tablespoonfuls of mustard, and rub the chest with warm camphorated oil.

### BREAD AND DYSPEPSIA.

The conclusion that wheat bread is unfit for dyspeptics, sometimes jumped at because ill effects are noticed to follow its use, is erroneous. On the contrary, it has been pointed out by Bouchard and others that farinaceous food is peculiarly adapted to some dyspeptic patients. It is the microbes in the starch, which is capable of producing irritating acids, that cause the trouble. To avoid this, Bouchard recommends that only the crust or toasted crumbs of the bread be used by dyspeptics, particularly those whose stomachs are dilated. The reason for this is explained by the fact that baking temporarily, though not permanently, arrests the fermentation of dough. When it is again heated by the warmth of the stomach the fermentation is renewed. In cases where the bread is toasted brown through the fermentation is stopped permanently.

### CARE OF THE EYEBROWS.

Scanty eyebrows and lashes spoil the appearance, and more especially when the hair is a lovely auburn or bronze brown, usually accompanied with blonde eyebrows, which appear at night almost colorless. Here the greatest mistake is made, as not infrequently the eyebrows are touched up with kohol or other black pigments, which are so palpably unnatural as to spoil the appearance utterly and suggest a liberal use of cosmetics.

A little harmless dye, or a blonde pencil applied with the lightest hand, will remedy the washed-out effect and appear perfectly natural; but those who have fairly marked eyebrows should not attempt anything of the kind, as nature is always preferable to art.

### CORRECT SITTING.

Many persons have formed the unhygienic and unphysiological habit of sitting on the spine, that is, sliding down in their chair in such a manner as seemingly to be sitting on the spine. Every time they sit down they take this unnatural position, throwing all the internal organs out of place, cramping them and causing congestion, etc., of all the parts. Now, after the habit is once formed, it is just as easy to sit correctly as incorrectly. If, when sitting down one will remember to press the hips well back in the chair, he will soon overcome not only this slouchy, unbecoming and reprehensible habit of sitting, but he will also break up another almost universal habit : that of bending the body forward at pit of stomach. If the hips are well back against the chair, you will find that you not only have but little inclination to bend forward at stomach, but that it will be almost impossible to do so.

### HOW TO MAKE AN INVALID'S BED.

The difficult problem of how to make a bed with a sick person in it is here solved. If the invalid's apparel is to be changed attend to that first ; then allow a little time to rest. Placing the patient on one side of the bed, with a light covering over her, proceed to make the other side, moving away the soiled lower sheet, putting on a clean sheet, folded in the middle of the bed lengthwise ; place a clean pillow ready for the head. Now move the patient over to the fresh side and make the other, drawing out the folded part of the sheet. Take the clean upper sheet and spread over the covering already on the bed. If the patient is not too ill to hold the upper part of the sheet she can do so ; if she is, pin each upper corner to the bed and from the foot draw out the loosened soiled sheet, or whatever it is to be removed, and put on the other covering.

### ANIMAL FOOD.

When we have active exercise in the open air, we may with impunity eat a hearty dinner ; taking care, even then, to leave off before the stomach is filled. But on days when persons of weak digestion do not go out of doors, and especially, when the mind has not been energetically occupied, it would be well to abstain altogether from solid animal food, and satisfy themselves with simple farinaceous matters, in the composition of which care should be taken that eggs are as sparingly used as possible. Nothing is a grosser blunder than that eggs are eligible for weak digestion, and for the diet of the sick. They never assimilate with the contents of a disordered stomach, but partly coagulate and form various crudities ; and partly generate a noxious vapor which under its real character of sulphureted hydrogen rises from the stomach into the mouth. The coloring material of an egg is sulphur.

### HOT MILK.

Hot milk is a most nutritious beverage—a real luxury the real value of which but few people know. Many why have abundance of milk never think of using it as a drink. A drink, did we say ? That's a mistake. We should eat milk instead of drinking it. That is, take it in small sips. Why? Because the casein of the milk, when it comes in contact with the acid of the gastric fluid, coagulates and forms curds, and if swallowed in large quantities at once, a large curd is formed which the stomach handles with difficulty. The gastric fluid can mingle much more readily with the small curds that result from sipping the milk.—*The Dietetic Gazette.*

### WET FEET AND COLDS.

The best way to overcome susceptibility to taking cold from getting the feet wet is as follows. Dip the feet in cold water, and let them remain there a few seconds. The next morning dip them in again, letting them remain in a few seconds longer ; the next morning keep them in a little longer yet, and continue this till you can leave them in half an hour without taking cold. In this way a person can become accustomed to the cold water, and he will not take cold from this cause. But be it understood that the " hardening " must be done carefully.

### FOR TENDER FEET.

For painful sore feet caused by excessive walking, long standing, or constant movement, as in the use of the sewing machine, a dusting-powder of equal parts of precipitated chalk and tannin, or the tannin alone, will be of much service. Apply twice daily, after bathing the feet in warm water.

### HOW TO MAKE EYE-WATER.

The simplest and one of the best eye-waters is made by putting ten grains of white vitriol into half a pint of elder or rosewater. Put a couple of drops in the eye, under the lids, morning and evening. If it stings too much, add more of the rose or elder water.

### THE LAWS OF HEALTH.

The true secret of health and long life lies in very simple things.

Don't worry.

Dont hurry. " Too swift arrives as tardy as too slow."

" Simplify ! Simplify ! Simplify !"

Don't overeat. Don't starve. " Let your moderation be known to all men."

Court the fresh air day and night. " O, if you knew what was in the air !"

Sleep and rest abundantly. Sleep is nature's benediction.

Spend less nervous energy each day than you make.

Be cheerful. "A light heart lives long."

" Work like a man ; but don't be worked to death."

Avoid passion and excitement. A moment's anger may prove fatal.

Associate with healthy people. Health is contagious as well as disease.

Don't carry the whole world on your shoulders, far less the universe. Trust the eternal.

Never despair. "Lost hope is a fatal disease."

Why she had a tired feeling.—Farmer (to medical man)—"If you get out my way, doctor, any time, I wish you'd stop and see my wife. She says she ain't feeling well."

Physician—"What are some of her symptoms?"

Farmer—"I dunno. This morning after she had milked the cows and fed the pigs and got breakfast for the laborers and washed the dishes and built a fire under the copper in the wash-house and done a few odd jobs about the house, she complained of feeling tired-like. I shouldn't be surprised if her blood was out of order. I fancy she needs a dose of medicine."—*Truth.*

The short, loose dress used for the gymnasium, worn even for an hour, would give some women a new idea of liberty, and would dispel many mistaken notions of helpnessness.

